# High Immunogenicity of a T-Cell Epitope-Rich Recombinant Protein Rv1566c-444 From *Mycobacterium tuberculosis* in Immunized BALB/c Mice, Despite Its Low Diagnostic Sensitivity

**DOI:** 10.3389/fimmu.2022.824415

**Published:** 2022-02-21

**Authors:** Xiuli Luan, Xueting Fan, Ruihuan Wang, Yunli Deng, Zixin Chen, Na Li, Yuhan Yan, Xiaoyan Li, Haican Liu, Guilian Li, Kanglin Wan

**Affiliations:** ^1^ State Key Laboratory for Infectious Disease Prevention and Control, National Institute for Communicable Disease Control and Prevention, Chinese Center for Disease Control and Prevention, Beijing, China; ^2^ Community Health Management Service Center, Longgang District Peoples Hospital of Shenzhen, Shenzhen, China; ^3^ Department of Infection Control, Longgang District People’s Hospital of Shenzhen, Shenzhen, China; ^4^ National Institute for Viral Disease Control and Prevention, Chinese Center for Disease Control and Prevention, Beijing, China; ^5^ Laboratory Animal Center, Chinese Center for Disease Control and Prevention, Beijing, China

**Keywords:** *Mycobacterium tuberculosis*, vaccine, antigen, diagnosis, T-cell epitope, Rv1566c

## Abstract

The discovery of immunodominant antigens is of great significance for the development of new especially sensitive diagnostic reagents and effective vaccines in controlling tuberculosis (TB). In the present study, we targeted the T-Cell epitope-rich fragment (nucleotide position 109-552) of Rv1566c from *Mycobacterium tuberculosis* (MTB) and got a recombinant protein Rv1566c-444 and the full-length protein Rv1566c with *Escherichia coli* expression system, then compared their performances for TB diagnosis and immunogenicity in a mouse model. The results showed that Rv1566c-444 had similar sensitivity with Rv1566c (44.44% Vs 30.56%) but lower sensitivity than ESAT-6&CFP-10&Rv3615c (44.4% Vs. 94.4%) contained in a commercial kit for distinguishing TB patients from healthy donors. In immunized BALB/c mice, Rv1566c-444 elicited stronger T-helper 1 (Th1) cellular immune response over Rv1566c with higher levels of Th1 cytokine IFN-γ and IFN-γ/IL-4 expression ratio by ELISA; more importantly, with a higher proliferation of CD4^+^ T cells and a higher proportion of CD4^+^ TNF-α^+^ T cells with flow cytometry. Rv1566c-444 also induced a higher level of IL-6 by ELISA and a higher proportion of Rv1566c-444-specific CD8^+^ T cells and a lower proportion of CD8**^+^** IL-4**^+^** T cells by flow cytometry compared with the Rv1566c group. Moreover, the Rv1566c-444 group showed a high IgG secretion level and the same type of CD4+ Th cell immune response (both IgG1/IgG2a >1) as its parental protein group. Our results showed the potential of the recombinant protein Rv1566c-444 enriched with T-Cell epitopes from Rv1566c as a host T cell response measuring biomarker for TB diagnosis and support further evaluation of Rv1566c-444 as vaccine antigen against MTB challenge in animal models in the form of protein mixture or fusion protein.

## Introduction

Tuberculosis (TB) caused by *Mycobacterium tuberculosis* (MTB) is a serious infectious disease, causing 1.51 million deaths worldwide in 2020 (1). The lack of universal health coverage, increasing drug resistance, and poor funding pose great barriers to ending TB. Rapid point-of-care diagnostic tests, new vaccines or effective preventative treatment, and safer, simpler, and shorter drug regimens are priorities to end TB epidemic.

In recent years, immunological methods have played an increasingly important role in the diagnosis of TB or latent TB infection (LTBI), and many biomarkers such as cytokines, antigens, and antibodies have been studied to use in these kinds of methods (2–4). Interferon-γ release assays (IGRAs) are tests used for evaluating cell-mediated host immune response according to T cells that release IFN-γ or the concentration of IFN-γ after stimulation by some of the RD1−encoded antigens (eg, the 6 kDa early secretory antigenic target [ESAT-6] and culture filtrate protein 10 [CFP-10] or TB 7.7) (5), and IGRAs are usually used to diagnose TB or LTBI, and as tools to identify new and more prominent antigen biomarkers. Although the present IGRAs have advantages in improving the specificity for diagnosing MTB infection in populations with late or repeated BCG vaccination or exposed to non-tuberculous mycobacteria, they fail to accurately differentiate between LTBI and active TB, their diagnostic accuracy also needs to be improved. Furthermore, new methods based on new antigens for effectively differentiating between LTBI and active TB are also needed (6). In the present study, we evaluated the performance of a designed IGRA with a new molecular to diagnose MTB infection.

*Bacillus Calmette-Guérin* (BCG), the only licensed vaccine before 2021, has been proven to induce protective immunity against TB (7). Although BCG is invaluable in preventing active TB disease in children <5 years of age, the efficacy of BCG vaccination in children wanes over time with protection generally lasting up to 10 years, so there is a great need for new vaccines against TB. Strategies on developing new vaccines are to design BCG substitutes, or BCG priming-heterologous vaccine boosters for the prevention of active TB, or to be therapeutic vaccines. The new TB vaccines under clinical trials can be mainly classified into three platform types: whole-cell or lysates of mycobacteria derived vaccines (includes recombinant BCG), viral vector vaccines, and adjuvanted recombinant protein vaccines (both called subunit vaccines). Whether for recombinant BCG or subunit vaccines, it is important to find antigens with excellent immunogenicity.

Previous studies showed that the epitopes of MTB have implications for the development of immuno-diagnostic tests and subunit prophylactic vaccines, and some of the epitopes showed promising results (8). An assay based on RD1 selected epitopes has been reported to have higher diagnostic accuracy for active tuberculous in a clinical setting compared with commercially available assays based on RD1 overlapping peptides spanning the whole proteins. All of these assays employ ELISA or ELISPOT techniques (9). Chen et al. reported that eliciting antibodies against specific MTB capsular glycan epitopes may increase vaccine efficacy against TB (10).

Rv1566c (or RipD), a 24-kDa antigen from MTB was identified as a putative exported/extracellular protein and as a homolog of NlpC_p60, which was found in a mycobacteriophage and 11 mycobacteria species and showed similar pentapeptide repeats in the cell wall (11). The Rv1566c antigen has been reported to show strong binding to peptidoglycan (12). *Rv1566c* is also identical to the *BCG1619c* gene of *Mycobacterium bovis* BCG, which encodes a putative invasin-like exported protein and contains a domain of the NlpC/p60 family (13), such NlpC/p60 domains are present in several bacterial proteins and implicate in host cell invasion and (or) intracellular persistence for MTB to cause the disease (14). Rv1566c has been reported to have considerable performance in serological diagnosis of TB (15). T-Cell epitopes in Rv1566c had been predicted in our previous study (16) by using TEpredict and IEDB-AR which have been universally used in predicting B-Cell or T-Cell epitopes and have been successfully used in predicting ten MTB protein antigens used in subunit vaccine candidates (such as M72, H1, H4, H56, and ID93) (17). Our predicted results showed that a total of 29 promising immunogenic human T-Cell epitopes were found in Rv1566c. In our previous study, the 29 epitope-located region was truncated into six peptides, then the peptides were synthesized and evaluated on immune reactivity and diagnosis performance. Of the six peptides, only one was confirmed to show immune reactivity and show a sensitivity of 5.8% and a specificity of 100% for TB diagnosis by ELISpot (16). Considering the immune reactivity and immunogenicity of a single peptide may not represent the whole epitode-rich region, we conducted the present study with a constructed protein that contained all of the 29 epitopes.

In the present study, we first intercepted a 444 bp sequence fragment in *Rv1566c* which contained all 29 predicted T-Cell epitopes of Rv1566c and started from 109 to 552 nucleotide position. Second, we had the purified protein (named Rv1566c-444) encoded by the chosen 444 bp sequence from *Escherichia coli* expression system. We then verified the possibility that Rv1566c-444 could replace the full-length protein Rv1566c as an effective antigen for detection of MTB infection in human population and show more prominent immunogenicity than Rv1566c in mouse models.

## Materials and Methods

### Ethics Statement

This study complied with the Ethics Committee of the National Institute for Communicable Disease Control and Prevention, Chinese Center for Disease Control and Prevention (No. ICDC-2019001). Each potential participant was introduced to the nature of the research and provided with an information sheet. Participants were included in this study if their written informed consent was obtained.

The animal experiments were conducted in accordance with the guidelines of the Animal Experimental Ethical Committee of the Chinese Center for Disease Control and Prevention (No. 2019-CCDC-IACUC-011). Animals received free access to water and commercial mouse chow and were monitored carefully throughout the study. The suffering of the mice was minimized during animal immunization and surgery procedures.

### Prediction of T-Cell Epitopes and Selection of T-Cell Epitope-Rich Sequences

**Figure 1:**
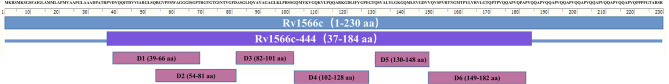
T -Cell epitope-rich domains distributed in Rv1566c. The scale unit is amino acid (aa).

The sequence of *Rv1566c* was obtained from the National Centre for Biotechnology Information (NCBI) at www.ncbi.nlm.nih.gov. T-Cell epitopes in Rv1566c binding to HLA-A02 supertype allele (including HLA-A*0201, *0202, *0203, *0206 motifs) were predicted by TEpredict and the tools available in IEDB-AR (http://tools.iedb.org/mhci/). T-cell pMHC class I immunogenicity predictor (http://tools.immuneepitope.org/immunogenicity/) was then used to evaluate the epitopes’ immunogenicity. In the present study, we chose a sequence enriched with the T-Cell epitopes of Rv1566c. This targeted DNA sequence was named Rv1566c-444n and is at the nucleotide positions from 109 to 552 in *Rv1566c*, the corresponding protein was defined as Rv1566c-444. The position details for Rv1566c-444 from Rv1566c are shown in **Table 1**, the distributions of all 29 predicted epitopes in the Rv1566c and Rv1566c-444 were located in six domains, as shown in **Figure 1**.

**Table 1:** Nucleotide and Amino acid positions of the selected Rv1566c-444 enriched with T-cell epitopes.

	Start at	End at	Length
Nucleotide sequence	109	552	444 bp
Amino acid sequence	37	184	148 aa

### Gene Amplification, Expression, and Purification of Recombinant Proteins pET- 32a-Rv1566c and pET-32a-Rv1566c-444

The whole sequence of *Rv1566c* and the targeted sequence Rv1566c-444n were amplified by polymerase chain reaction (PCR) with H37Rv genome DNA as a template. The sequences were then respectively ligated into plasmid pET-32a after digestion with EcoRI and HindIII and then transformed into *E. coli* BL21 (DE3). One mM isopropyl β-D-thiogalactoside (IPTG) was used in the medium to induce the expression of the proteins. After incubating for 3.5 h at 37°C with shaking the cells were harvested by centrifugation (4,000 rpm, 10 min, 4°C). The expression level and form of Rv1566c and Rv1566c-444 in *E. coli* were evaluated by 12% sodium dodecyl sulfate-polyacrylamide gel electrophoresis (SDS-PAGE), then the inclusion body proteins were denatured and used for protein purification by Ni-NTA chromatography (GE Healthcare, Pittsburgh, PA, USA), followed by renatured in 20mM Tris-HCL (pH 8.0). Endotoxin from the recombinant protein was removed using a ToxinEraser endotoxin removal kit (GenScript, Piscataway, NJ, USA) and then the solution was sterile filtered using a 0.22-mum filter followed by quantification with a bicinchoninic acid kit (Transgen Biotech, Beijing, China). The preparation of Ag85B was done as described previously by our laboratory colleagues (18, 19).

### Participant Collection

Participants included TB patients and healthy donors who were recruited during June 2019 in Beijing for the ELISpot assay. All of the subjects were vaccinated with *M. bovis* BCG before. The TB patients were used to represent the population actively infected with MTB. All TB cases were in compliance with the national criteria for the TB diagnosis in China: having a positive acid-fast smear or MTB culture or MTB gene test, and a chest x-ray suggestive for having active TB infection. The healthy controls were randomly selected among healthy volunteers without clinical TB symptoms or signs and conscious of prior contact history to MTB. All of the controls did not receive tuberculin skin tests in the present study. We excluded individuals taking immunosuppressive agents or pregnant women.

### Measurement of Rv1566c-444 Specific IFN-γ Production by ELISpot in Human Donors

A commercial ELISpot kit T-SPOT.TB (QuanBio, Beijing, China) was used to detect the T- cells producing IFN-γ according to the instructions of the manufacturer. The PBMCs from each human participant were prepared by Ficoll–Hypaque density gradient centrifugation and suspended in an AIV medium. One hundred microliters (2.5×10^5^ cells) per well of PBMCs were seeded in 96-well nitrocellulose plates precoated with the anti-IFN-γ monoclonal antibody and then stimulated with either a cocktail of peptides provided in the kit including ESAT-6, CFP-10, and Rv3615c or antigens prepared in this study Rv1566c-444 (20 μg/mL) or Rv1566c (20 μg/mL). Phytohemagglutinin (PHA) was used as a positive control and AIV medium as a negative control. Spot forming cells (SFCs) were counted after 20 h incubation at 37°C. Spot forming cells representing the T cells releasing IFN-γ were used to compare the diagnosis performance for MTB infection between Rv1566c-444, Rv1566c, and T-SPOT.TB (QuanBio, Beijing, China).

### Mice and Immunization Schedule

Specific-pathogen-free and female BALB/c mice were purchased from Beijing HFK Bioscience Co.127 Ltd. (Beijing, China) and were age-matched (6 weeks) within each experiment. Five groups of six mice each were vaccinated.

The DP mixture composed of DDA and Poly I: C was used as an adjuvant in this study. DDA is known as a good adjuvant that facilitates antigen adsorption and presentation, which can induce a mixed Th1/Th2 immune response with the predominance of Th1 cytokines when vaccinated in BALB/c mice (20). Poly I: C is a ligand of Toll-like receptor 3 (TLR3) which can attenuate the pathologic reaction induced by MTB and can induce innate immunity and Th1 cytokine production (21). DP mixture was prepared by mixing DDA (2.5 mg/mL) and poly I: C (0.5 mg/mL) in Tris-HCl (pH 8.0), the vaccines were prepared with the antigens (100 μg Rv1566c-444, Rv1566c, or Ag85B) emulsified in the same volume of DP mixture. All mice were vaccinated subcutaneously with the emulsion on the 1st, 14th, and 28th day. The positive control mice were immunized with Ag85B, and the negative control groups were given 200 μL Tris-HCl (pH 8.0) or 200 μL DP mixture. The BALB/c mice were sacrificed for immunogenicity assessment at 35th day after the first immunization.

### Humoral Immunogenicity of Rv1566c-444 and Rv1566c in BALB/c Mice

The specific total IgG and IgG subclass titers in serum were determined by ELISA assay to detect the humoral responses against Rv1566c-444, Rv1566c, or Ag85B vaccinations. Ninety-six well ELISA plates were first prepared in triplicate by adsorbing with the protein Rv1566c-444, Rv1566c, or Ag85B diluted with coating buffer and the final concentrations of the proteins were all 20 μg/mL. After incubation overnight at 4°C, the plates were washed 3 times with 0.01 M phosphate-buffered saline (PBS, pH 7.4) containing 0.05% Tween-20 (PBST) followed by blocked with 100 μL of blocking solution contained 3% BSA for 2 h at 37°C. After the blocking step, serum samples from the mice vaccinated with Rv1566c-444, Rv1566c, or Ag85B were diluted doubly in PBS and were added in the plate wells containing corresponding antigens, and incubated again at 37°C for 1 h. After that, the plates were washed five times with PBST, then horseradish peroxidase-labeled goat anti-mouse IgG and IgG subclass antibody were diluted (1:1000) and added to the plates (100 μL/well). Following repeated washes, 100 μL of Tetramethylbenzidine substrate was added to each well and incubated for 15 min at room temperature. The color reaction was terminated by adding 50 μL H_2_SO4 (2 M). Finally, the optical density for each sample was read using an ELISA reader at 450 nm and expressed as absorbance.

### Cellular Immunogenicity of Rv1566c-444 and Rv1566c in BALB/c Mice

After collecting blood by retro-orbital puncture, the mice were sacrificed by cervical dislocation, the spleens were aseptically removed from the mice and used for splenocyte harvest after grinding the spleens and lysis of erythrocytes. The splenocytes were diluted to 2×10^6^ cells/mL for cytokine testing and determination for the proportion of CD4^+^ T and CD8^+^ T cells. Every test was performed in triplicate.

First, ELISA was used to detect the cytokines after the splenocytes were stimulated with the vaccine antigens. Cells stimulated with concanavalin A (ConA) were used as positive, and cells in RPMI 1640 media without antigen as the negative control. After incubation at 37°C with 5% CO_2_ for 48 h, the supernatants were collected and assayed for the presence of interferon (IFN)-γ, interleukin (IL)-4 and IL-6 by commercial ELISA kits (BD, San Diego, CA, USA) according to the manufacturer’s instructions.

Second, flow cytometry on a FACScalibu and selected agents (all from BD, San Diego, CA, USA) were used to detect the proportions of CD4^+^ T and CD8^+^ T cells, and the proportions of cytokine secreting CD4^+^ T or CD8^+^ T. After being cultured with protein transport inhibitor and recall antigens (Tris-HCl and DP groups both used Rv1566-444) for 8 h at 37°C in 5% CO_2_, the splenocytes were stained with LIVE/DEAD Fixable Dead Cell Stain, followed by surface staining with anti- CD3, CD4, and CD8. Then the cells were stained intracellularly with antibodies against IFN-γ, TNF-α, and IL-4. Finally, the splenocytes were fixed in 4% paraformaldehyde and detected by flow cytometry.

### Statistical Analysis

The experimental data were expressed as the mean ± SD using GraphPad Prism 8. One-way analysis of variance (ANOVA) with Turkey’s multiple comparison tests were used for data of more than two groups, *P-values <*0.05 were considered to be statistically significant. The receiver operating characteristic (ROC) curve was used to evaluate the performance of the biomarkers used in ELISpot in discriminating two categories (active MTB infection and health) with the MedCalc statistical software. The differences between the areas under the curve (AUCs) were compared using the pairwise comparison of ROC curves, *P*<0.05 meant the differences were statistically significant.

## Results

### Expression and Purification of the Recombinant Proteins

**Figure 2:**
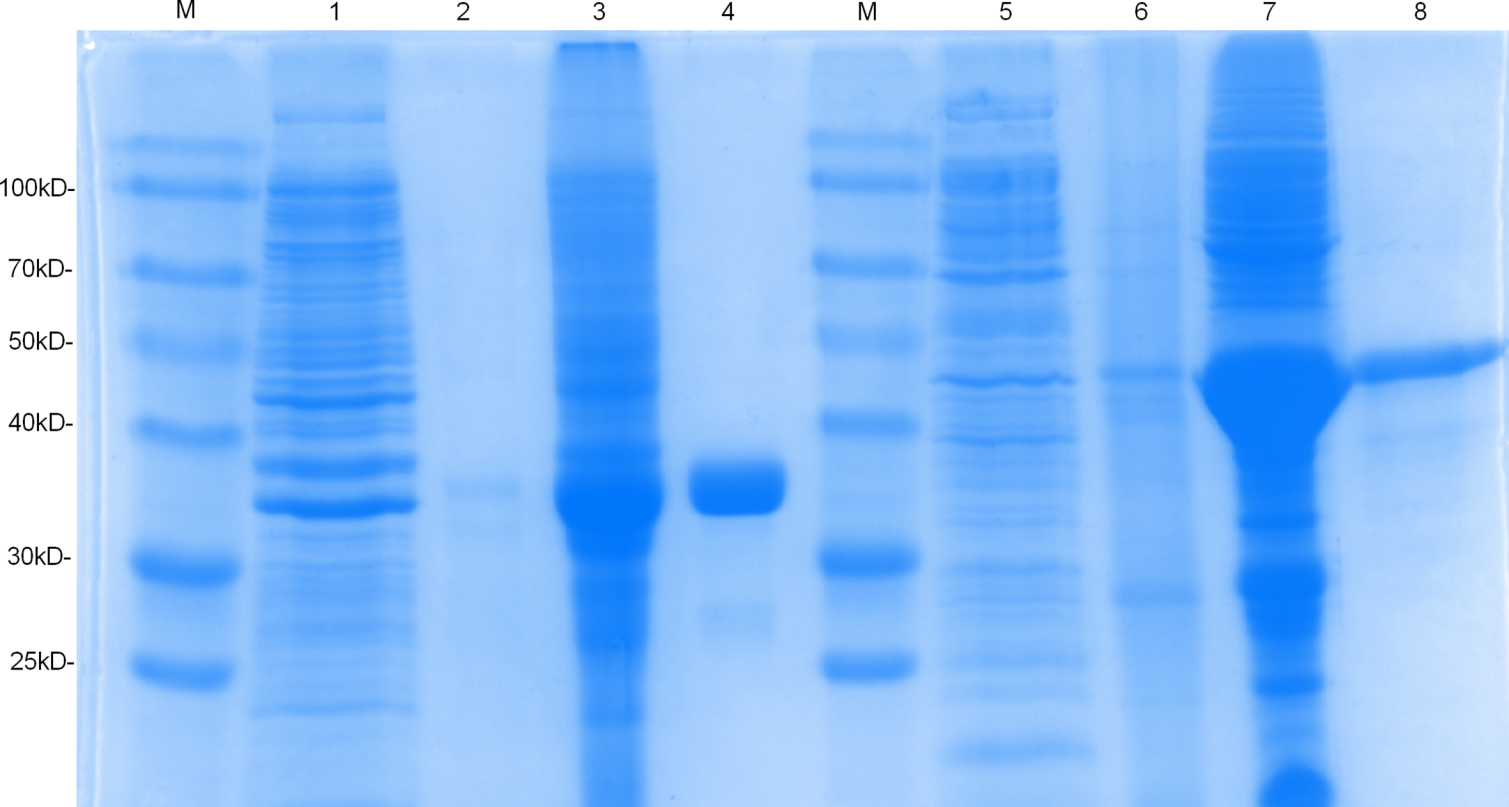
SDS-PAGE analysis of the purification and expression of recombinant protein Rv1566c-444 and Rv1566c. Lane M, Prestained protein marker; Lane 1, the uninduced pET32a-Rv1566c-444 transformed *E. coli*; Lanes 2 and 3, the supernatant and sediment of the induced pET32a-Rv1566c-444 after ultrasonication and centrifugation; Lanes 4: purified recombinant protein Rv1566c-444; Lanes 5, the uninduced pET32a-Rv1566c transformed *E. coli*; Lanes 6 and 7, the supernatant and sediment of the induced pET32a-Rv1566c after ultrasonication and centrifugation; Lanes 8: purified recombinant protein Rv1566c.

The sequences Rv1566c-444n and *Rv1566c* were successfully cloned into the pET32a plasmid respectively and confirmed by PCR and DNA sequencing. As shown in **Figure 2**, the SDS-PAGE analysis indicated that the Rv1566c-444 and Rv1566c proteins were mainly expressed in the form of inclusion body proteins and showed about 35 and 45 kDa proteins respectively which were in line with the predicted results.

### Characteristics of the Participants

Overall, 80 participants, including 36 patients with pulmonary TB from Beijing Changping Institute for Tuberculosis Prevention and Treatment, and 44 healthy donors from the Chinese Center for Disease Control and Prevention, Beijing, China were enrolled for the study. The TB patients comprised of 58.33% (21/36) men and 41.67% (15/36) women and aged from 20 to 76 years with an average age of 39.44 ± 15.73 years; the healthy donors comprised 36.36% (16/44) men and 63.64% (28/44) women and aged from 22 to 38 years with an average age of 26.61 ± 5.36 years.

### Rv1566c-444 Specific IFN-γ Production in Human Donors

**Figure 3:**
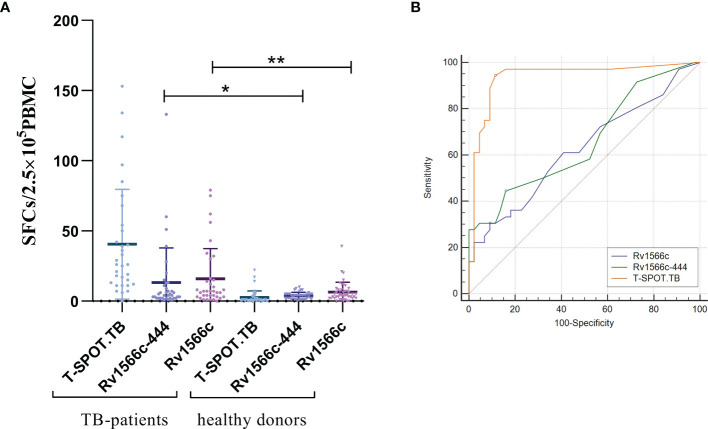
Analysis of the IFN-γ ELISpot response to the recombinant protein Rv1566c-444, Rv1566c in tuberculosis patients and healthy donors. **(A)** The difference of IFN-γ SFCs in Rv1566c-444, Rv1566c, and T-SPOT. Each dot represents the SFCs of one patient or donor. The average response in each group is indicated by solid lines. The *P-values* of the differences in SFCs between the groups were determined by one-way ANOVA with Turkey’s multiple comparison tests (*p < 0.05, **p < 0.01). **(B)** ROC curves analysis of Rv1566c-444, Rv1566c, and cocktail peptides in T-SPOT kit for ELISpot.

To evaluate the diagnostic value of cellular immunity of Rv1566c-444, IFN-γ-producing effector T cells in PBMCs (presented as SFCs) from TB patients and healthy donors were detected by ELISpot. As shown in **Figure 3A**, both Rv1566c-444 and Rv1566c induced higher SFCs in TB patients than in healthy donors (*P*<0.05 and *P*<0.01), while there was no difference between the SFCs induced by Rv1566c-444 and Rv1566c in TB patients, which was consistent with the hypothesis that T-Cell epitope-rich fragment induces similar IFN-γ production in T cells with its parental protein. The AUC of T-SPOT.TB was significantly higher than those of Rv1566c-444 and Rv1566c (*P* values were all*<*0.0001, **Figure 3B** and **Table 2**), while no statistical significance between Rv1566c-444 and Rv1566c was found. We also found that humans with a cut-off value of SFC>5 (sensitivity, 44.44%; specificity, 84.09%) induced by Rv1566c-444 were diagnosed as MTB infection (**Table 2**).

**Table 2:** Evaluation results from the ROC curve of the recombinant proteins for active *Mycobacterium tuberculosis* infection diagnosis through ELISpot assay.

Antigens	Youden index	Sensitivity (%)	Specificity(%)	AUC	*P*-value	Associated criterion
Rv1566c-444	0.3081	44.44	84.09	0.656	0.0119	>5 SFCs
Rv1566c	0.2146	30.56	90.91	0.624	0.0523	>13 SFCs
T-SPOT.TB	0.8308	94.44	88.64	0.937^*^	<0.0001	>6 SFCs^#^

ROC curve, receiver operating characteristic (ROC) curve; SFCs, spot forming cells; AUC: the areas under the ROC curve. According to the pairwise comparison results of the ROC curve, for the T cell counts acquired by the ELISpot assay, *the AUC of T-SPOT.TB was significantly higher than those of the other two recombinant proteins (Rv1566c-444 and T-SPOT.TB: z = 4.244, P < 0.0001; Rv1566c and T-SPOT.TB: z = 4.719, P < 0.0001), no statistical significance was found between Rv1566c-444 and Rv1566c. ^#^This criterion was acquired by the ROC curve, while according to the manufacturer’s instructions, the criterion for M. tuberculosis infection (not only active infection) is as follows: The results were considered to be positive if either the panel test yielded ≥ 6 SFCs after subtracting the background spots of the negative control well when the negative control had ≤ 5 SFCs, or if the number of spots in the panel test was at least double that of the negative control which with SFCs ranged from 6 to 10, and negative in any other case with SFCs in positive control was less than 20.

### Comparison of Humoral Immunogenicity Between Rv1566c-444 and Rv1566c in Mouse Model

**Figure 4:**
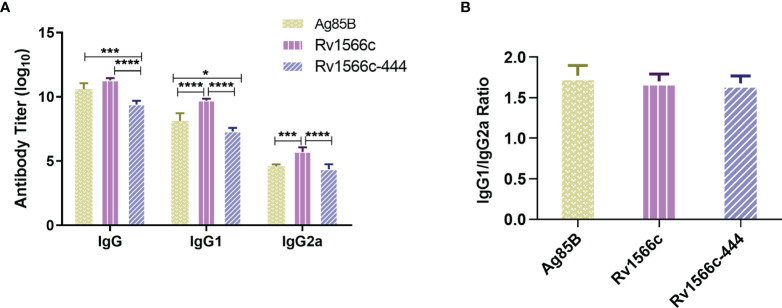
Assays of IgG, IgG1, and IgG2a isotypes in sera of Rv1566c-444, Rv1566c, and Ag85B immunized BALB/c mice. **(A)** Antibody titers of total IgG, IgG1, and IgG2a were compared between the three groups. **(B)** Antigenic IgG1/IgG2a ratios were compared between the three groups. The *P-values* of the differences were determined by one-way ANOVA with Turkey’s multiple comparison tests (*P < 0.05, ***P < 0.001, ****P < 0.0001).

To verify whether Rv1566c-444 induces a high humoral response, the quantities of serum IgG, IgG1, and IgG2a isotypes specific to Rv1566c-444 were compared to those specific to Rv1566c and Ag85B by ELISA assays. As Tris-HCl immunized group and DP group had similar and low absorbance by the ELISA assay, we chose the former group as the negative control. As shown in **Figure 4A**, all of the three protein-adjuvanted vaccines elicited significantly higher titer of specific IgG, IgG1, and IgG2a compared to the negative control. Among the three protein-adjuvanted vaccinated groups, Rv1566c-444 induced lower levels of IgG, IgG1, and IgG2a than Rv1566c (*P* values were all < 0.0001) and lower levels of IgG and IgG1 than Ag85B (*P* values were <0.001 and <0.05, respectively) (**Figure 4A**); though there was no statistical significance on the levels of IgG between Rv1566c and Ag85B, the IgG1 and IgG2a levels in Rv1566c group were both higher than in Ag85B group. There was no statistical difference among the ratios of IgG1 to IgG2a induced by Rv1566c-444, Rv1566c, and Ag85B with ratios of 1.678, 1.701, and 1.711, respectively (**Figure 4B**).

### Rv1566c-444 Induced Stronger CD4 Th1 and CD8 T Cell Response in Mice

**Figure 5:**
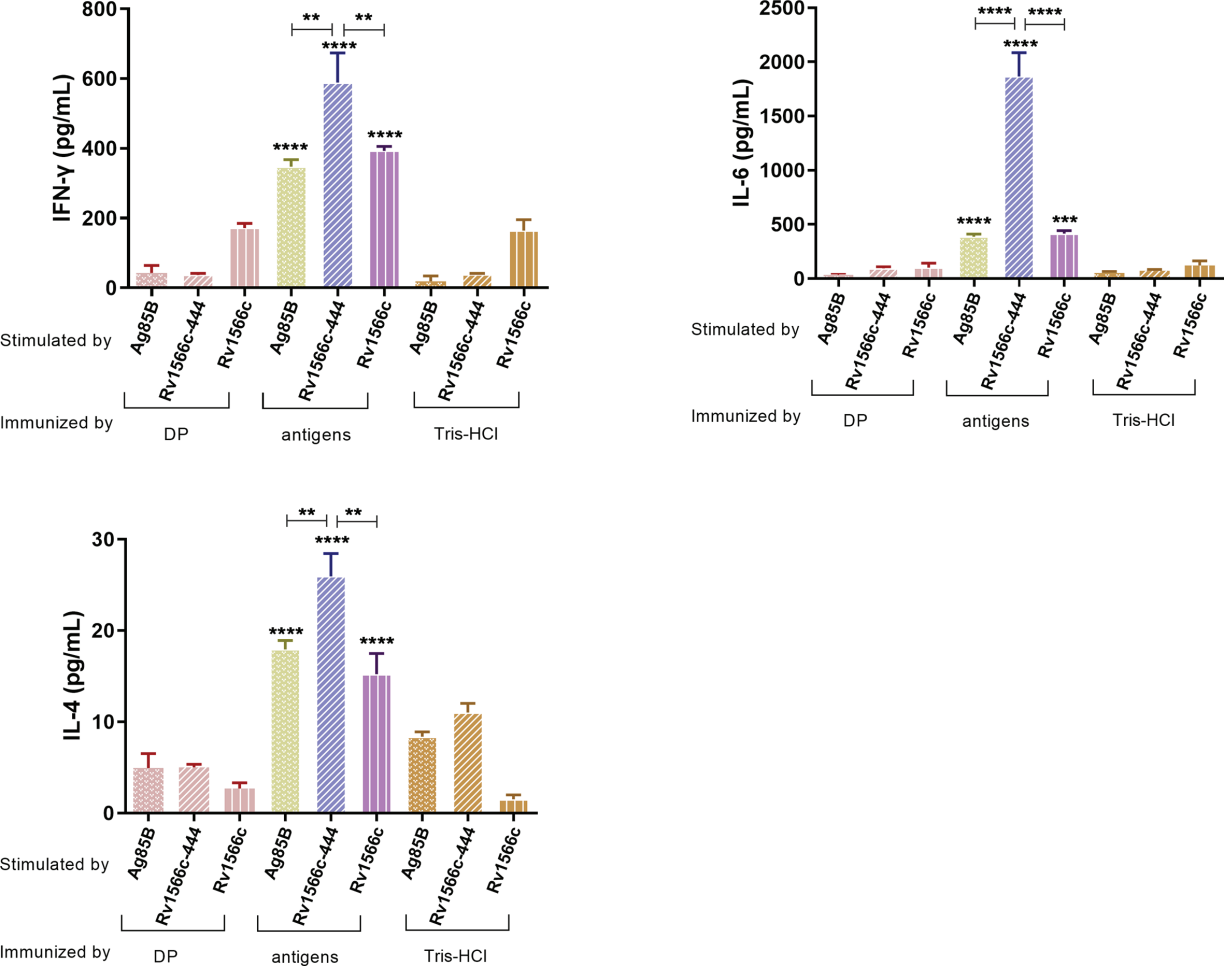
Antigen-specific cytokine levels were released from the splenocytes of the immunized mice. After lymphocytes were isolated from BALB/c mice immunized with the constructed vaccines, they were stimulated with antigens used in the vaccines respectively. BALB/c mice immunized with DP mixture or Tris-HCl were also stimulated with three antigens respectively. The concentration of three different cytokines **(A)** IFN-γ, **(B)** IL-6, and **(C)** IL-4 were measured by ELISA assays. The significant differences between antigen and negative controls (DP and Tris-HCl groups) both stimulated with the same antigen are represented by asterisks above the bar; significant differences among three antigens groups (Rv1566c-444, Ag85B, and Rv1566c) are presented by capped lines with asterisks. The statistical significance was determined using one-way ANOVA with Turkey’s multiple comparison tests (**P < 0.01, ***P < 0.001, ****P < 0.0001).

The levels of Th1-type cytokine IFN-γ and Th2-type cytokine IL-4 together with IL-6 produced by the recall antigens-stimulated spleen cells from the mice vaccinated with Rv1566c-444, Rv1566c, and Ag85B were evaluated and compared with those from Tris-HCl and DP adjuvant vaccinated groups. All of the groups except the Tris-HCl and DP groups showed high production of the three cytokines (**Figure 5**). A significant increase in the productions of IFN-γ and IL-4 upon stimulation with recall antigen was observed in Rv1566c-444 immunized mice compared with the Rv1566c and Ag85B groups (all *P values*< 0.01, **Figures 5A, C**). No significant difference in the concentrations of IFN-γ was observed between the Rv1566c group and Ag85B group (**Figure 5A**). A significantly higher level of IL-6 secretion was observed in the Rv1566c-444 group compared with the Rv1566c and Ag85B groups (all *P values*< 0.0001) (**Figure 5B**). Rv1566c-444 had a higher IFN-γ/IL-4 ratio than Rv1566c (25.61 *vs*. 23.10, *P*< 0.05), which indicated that the Rv1566c-444 induced stronger Th1 responses than Rv1566c.

**Figure 6:**
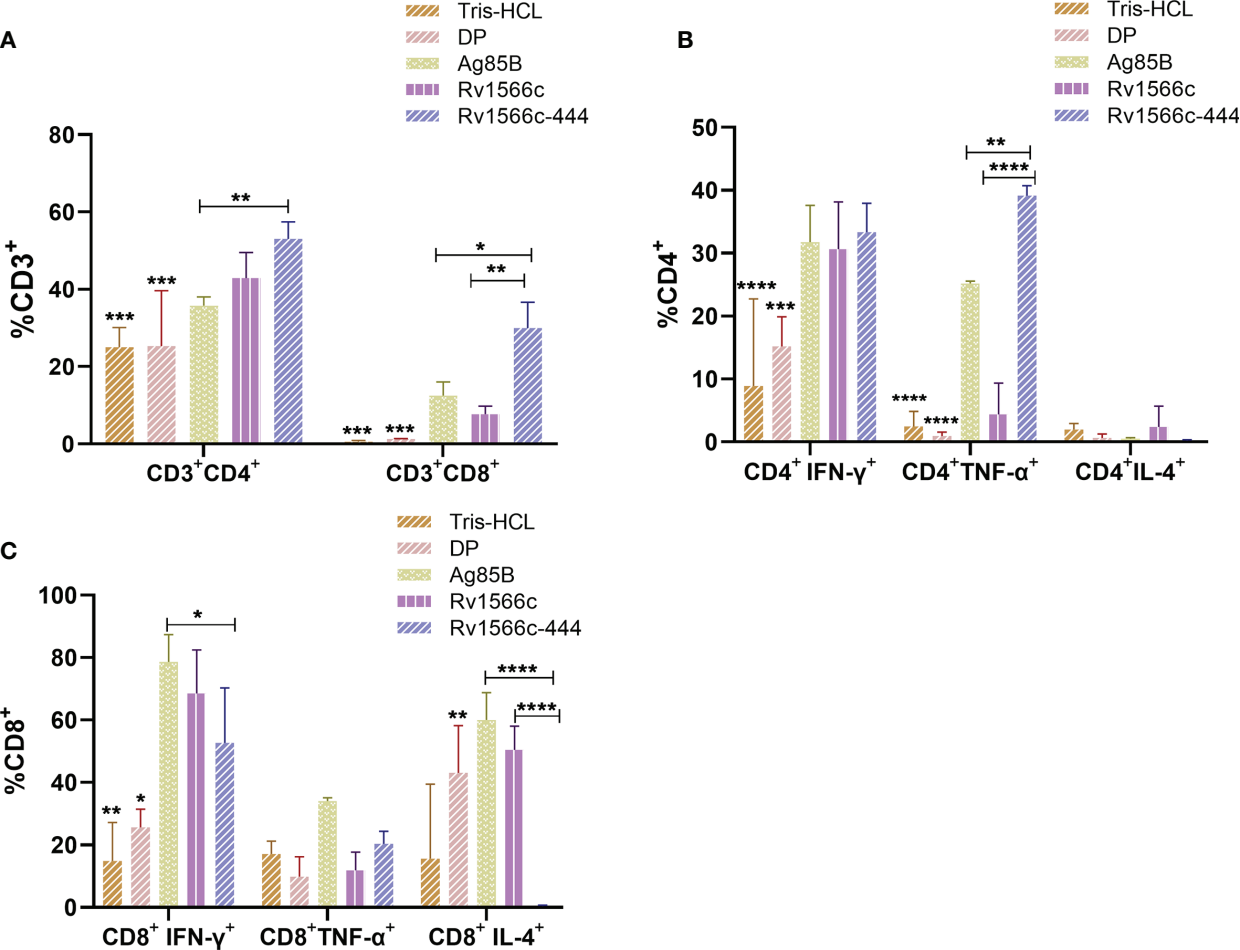
Proportions of CD3^+^ CD4^+^ T cells and CD3^+^ CD8^+^ T cells in spleen cells from mice immunized with different vaccines and proportions of CD4^+^ or CD8^+^ T cells that secreted different cytokines. Splenocytes from BALB/c mice immunized with protein vaccines were respectively stimulated with the protein used in each vaccine, DP adjuvants, and Tris-HCl were stimulated with Rv1566c-444. The proportions of cells were assessed by flow cytometry. **(A)** Proportions of CD3**^+^** CD4**^+^** T cells and CD3**^+^** CD8**^+^** T cells in spleen cells from mice immunized with different vaccines. **(B)** Proportion of CD4^+^ T cells producing IFN-γ/TNF-α/IL-4. **(C)** Proportion of CD8^+^ T cells producing IFN-γ/TNF-α/IL-4. The significant differences between the Rv1566c-444 group and negative controls (DP and Tris-HCl groups) both stimulated with Rv1566c-444 are represented by asterisks above the bar; significant differences among antigens groups were presented by capped lines with asterisks. The statistical significance was determined using one-way ANOVA with Turkey’s multiple comparison tests (*P < 0.05, **P < 0.01, ***P < 0.001, ****P < 0.0001).

We also analyzed the stimulated splenocytes using flow cytometry to detect the proportions of CD3**^+^** CD4**^+^** T cells and CD3**^+^** CD8**^+^** T cells in spleen cells together with the proportions of CD4**^+^** or CD8**^+^** T cells that secreted different cytokines. Our results showed that Rv1566c-444 immunized mice had significant higher proportions of CD3**^+^** CD4**^+^** T cells and CD3**^+^** CD8**^+^** T cells than the Tris-HCl and DP groups (all *P values* < 0.001), and had CD3**^+^** CD4**^+^** T cells that were significantly higher than those of Ag85B group (*P*< 0.01) and similar to those of Rv1566c group; Rv1566c-444 group showed significant higher percentage of CD3**^+^** CD8**^+^** T cells than Rv1566c (*P*< 0.01) and Ag85B groups (*P*< 0.05) (**Figure 6A**). Among the CD3**^+^** CD4**^+^** T cells, the Rv1566c-444 group had a significantly higher percentage of CD4^+^IFN-γ^+^ T cells than the Tris-HCl group (*P*< 0.0001) and DP group (*P*< 0.001), whilst no statistical difference was observed among Rv1566c-444, Rv1566c, and Ag85B groups with percentages of 33.33%, 30.63%, and 31.77%, respectively. A significantly higher proportion of CD4**^+^** TNF-α **^+^** T cells were observed in Rv1566c-444 immunized mice compared with the Ag85B group (*P*< 0.05) and the other groups (all *P values* < 0.0001) (**Figure 6B**). Among the CD3**^+^** CD8**^+^** T cells, we found that the proportion of CD8**^+^** T cells expressing IFN-γ in the Rv1566c-444 group was significantly higher than those in DP (*P*< 0.05) and Tris-HCl groups (*P*< 0. 01), significantly lower than that in Ag85B group (*P*< 0.05) and similar to that in Rv1566c group; the percentage of CD8**^+^** T cells expressing IL-4 in the Rv1566c-444 group was lower than those in the Rv1566c group (*P*< 0.05), Ag85B group (*P*< 0.01), and DP group (*P*<0.05), respectively (**Figure 6C**).

## Discussion

TB, although a curable and preventable disease, continues to be one of the leading infectious causes of death worldwide. The existence of antibiotic-resistant strains also emphasizes the need for the development of advanced diagnostic tools and a vaccine solution to curb the spread of TB. Identification of the immunodominant MTB antigens from the antigen pools induced host cell-mediated protective immunity is helpful for the development of MTB diagnosis tools or TB vaccines. In the present study, we evaluated the performance of the recombinant protein Rv1566c-444 enriched with T-Cell epitopes as a diagnostic biomarker of active MTB infection in humans and its immunogenicity in mice. This last assay could provide implications for using this recombinant protein as a vaccine antigen. Then we compared the antigenic properties of Rv1566c-444 to those of Rv1566c.

Rapid biomarker-based and non-sputum-based diagnostic tests able to accurately diagnose TB are in urgent need. They should be biosafe and appropriate for children, extrapulmonary TB patients, and individuals infected with the human immunodeficiency virus (HIV) who often have difficulty providing good-quality sputum samples. Among the current non-sputum-based tests, IGRAs are immunodiagnostic tests that rely on the detection of human T cell immune responses against mycobacterial antigens, some of them have been endorsed by the WHO (22). The WHO also provided an expanding pipeline of new IGRAs to test for TB infection (22). Neither IGRA nor tuberculin skin test (TST) is useful to distinguish LTBI from TB (23). Thus, developing a new generation of IGRAs with higher accuracy for MTB infection diagnosis or with the ability to discriminate LTBI and TB is still needed. It is generally accepted that latency antigens encoded by genes remaining up-regulated during LTBI identified by *in vitro* model and genome-wide transcriptome analysis have the potential to be immunological markers that could discriminate between LTBI and TB (23). Among a number of latency antigens, proteins belonging to DosR regulon, the Rpf family, and IVE-TB group have been studied as LTBI biomarkers (24, 25). Studies on the DosR and Rpf antigens from MTB to discriminate between latent and active TB in a TB endemic population of Medellin Colombia showed that some antigens of DosR regulon have the highest potential for differentiating LTBI from active TB (26). None of the evidence has shown that Rv1566c is a latency antigen or active TB antigen. In the present study, we only evaluated the performance of Rv1566c and Rv1566-444 to discriminate between healthy donors and TB patients. Our results showed that both Rv1566c-444 and Rv1566c induced higher SFCs in the TB patients than that in healthy donors, and there was no significant difference between the AUCs of Rv1566c-444 and Rv1566c, which indicated that the T epitopes-based protein Rv1566c-444 has the same antigenic properties as the whole Rv1566c protein in humans and can replace Rv1566c in the use for MTB infection diagnosis. We also observed that compared with ESAT-6&CFP-10&Rv3615c contained in the T-SPOT.TB kit, Rv1566c-444 had a far lower sensitivity (44.4% Vs. 94.4%) for distinguishing TB patients from healthy donors. As a single antigen, the diagnostic sensitivity of Rv1566c-444 was promising. More efforts can be made to combine Rv1566c-444 with other immunodominant antigen candidates to improve the diagnostic sensitivity.

By October 2021, there are 16 TB vaccine candidates under phase I - III clinical trials in the world (22, 27, 28), and Vaccae (Anhui Zhifei Longcom, China) was approved for use by the China Food and Drug Administration in June 2021. Among these vaccines, the recombinant BCG vaccine (VPM1002), the protein/adjuvant vaccines (such as M72/ASO1E, ID93+GLA-SE, AEC/BC02, and so on), and the viral vector vaccines (Ad5Ag85A, ChAdOx185A-MVA85A, and TB/FLU-04L) were all based on the peptides or proteins that show prominent immunogenicity and induced host immunity. It was reported that M72/ASO1E achieved a 49.7% (90% confidence interval [CI], 12.1 to 71.2%; 95% CI, 2.1 to 74.2%) efficacy at 36 months after vaccination among adults infected with MTB according to the phase IIb clinical trial results (29). However, none of these novel TB vaccines can be an alternative for BCG, which is recommended as part of national childhood immunization programs by the WHO. Vaccines providing longer protective time, higher efficacy and for different populations with negative MTB infection, LTBI, or TB under-treating are still needed. Meanwhile, the discovery of new immunodominant antigens is still an important prerequisite for the development of novel TB vaccines to break the limitations. In the present study, in addition to assessing and comparing the diagnosis potential between Rv1566c-444 and Rv1566c, we also evaluated their immunogenicity in mouse models and explored their potential to be vaccine antigens. The immunodominant antigen Ag85B was reported to induce cellular and humoral immunity responses in TB patients (30, 31). In mouse models, Ag85B was found to elicit a strong Th1 immune response against MTB challenge with an increased humoral IgG antibody production (32–34). Hence, the Ag85B antigen was used as a positive control in the present study for evaluating the immunogenicity of Rv1566c-444 in mice.

Though cell-mediated immunity has been proved to play the key role in protection from and clearance of infectious mycobacteria, humoral immunity was also reported to enhance human immunity against MTB infection in different ways: complexes of MTB bound to an antibody can be more readily phagocytosed by macrophages *via* crystallizable fragment receptors (FcRs) and complement receptors (35); antibodies specific for MTB can increase phagosome lysosome fusion, and the specific antibodies may stimulate killing of MTB infected cells *via* natural killer (NK) cell-mediated antibody-dependent cell cytotoxicity; antibodies against MTB may have direct microbicidal or neutralizing activity, and antibodies against MTB have been shown to promote inflammasome activation in macrophages. Besides, the protection against TB by antibodies also requires cell-mediated immune functions (35). Our results showed that Rv1566c-444 vaccination induced high-level titers of specific IgG, IgG1, and IgG2a, though the secretion levels were lower than those of the Rv1566c group, the reduced humoral immunity response of Rv1566c-444 may be due to the reduced numbers of B- Cell epitopes in Rv1566c-444 compared with Rv1566c. The Rv1566c-444 group aroused a similar secretion of IgG2a compared to Rv1566c and had similar ratios of IgG1/IgG2a compared with Rv1566c and Ag85B group. IgG2a is a Th1-type antibody, whilst IgG1 is a Th2-type antibody, the ratios of IgG1/IgG2a induced by Rv1566c-444 and Rv1566c vaccination in our study were floating around one indicating a Th2/Th1-type balance, suggesting that T-Cell epitope-rich fragment induces the same type of CD4^+^ Th cell immune response with its parental protein.

T cells are the primary mediators of adaptive immunity against TB, studies on mice and humans support the important roles of CD4^+^ T and CD8^+^ T cells in TB immunity (36, 37). The pre-clinical and under-clinical subunit protein TB vaccine candidates are inherently a CD4^+^ T cell-targeting platform with an emphasis target on protective multifunctional Th1 cells which are the major IFN-γ secreting cells in humans. Rodo et al’s study on six novel TB vaccine candidates MVA85A, AERAS-402, H1:IC31, H56:IC31, M72/AS01E, ID93+GLA-SE, and the licensed TB vaccine BCG showed that all vaccines preferentially induced antigen-specific CD4 T cell responses expressing Th1 cytokines; the response magnitude is the most differential characteristics among the vaccines (38). IFN-γ is an important host defense effector molecule in human that can inhibit inflammation during TB. It was reported that BCG vaccinated infants who developed disseminated BCG disease had mutations in genes encoding proteins involved in the IL-12/IFN-γ axis (39). IL-4, the archetypal T-helper type 2 (Th2) cytokine, can subvert mycobacterial containment in human macrophages, probably through perturbations in regulatory T-cell and Th1-linked pathways (40). Our results from the cytokine detection suggested that Rv1566c-444 inclined to induce Th1/Th2 imbalance response with a preference for a Th1 response based on the IFN-γ/IL-4 ratio, and generated the strongest Th1 cell-polarized CD4^+^ T cell responses (representing as the highest production of antigen-specific IFN-γ) among the three vaccines (**Figure 5A**). The results of flow cytometry also showed that the CD3^+^CD4^+^ proportion induced by Rv1566c-444 was significantly higher than that by Ag85B and was trending higher than that of Rv1566c, too. Additional cytokines are involved in protective immunity against TB and play roles in bacterial control after MTB infection. It was reported that mice with intact IFN-γ but a deficiency in IL-6 and TNF-α all succumbed to MTB after infection (41). Another report found that IL-6 and TNF-α as BCG induced pulmonary effector molecules may be more important than IFN-γ in IL-12 deficient and BCG-vaccinated mice (42). In our study, higher levels of IL-6 were induced by Rv1566c-444 than by Rv1566c and Ag85B respectively, suggesting that Rv1566c-444 as vaccine antigen may provide more immune protection.

Mittrücker et al. reported that the protection correlated best with the rapid accumulation of specific CD8^+^ T cells in infected tissues of challenged mice (43). CD8^+^ T cells play a critical role **i**n mediating immune protection against MTB: involving in the destruction of heavily infected macrophages and acting as a redundant effector of CD4^+^ T cells in cytolytic functions and IFN-γ secretion (44). According to our results acquired by flow cytometry, we observed that Rv1566c-444 induced a higher portion of CD8^+^ T cells but a far lower proportion of IL-4-secreting CD8^+^ T cells, meanwhile it induced a higher portion of CD4^+^ T cells with a far higher proportion of TNF-α secreting CD4^+^ T cells and a comparable proportion of IFN-γ secreting CD4^+^ T cells than Rv1566c in mice. Our results suggested that Rv1566c-444 induces a better CD4^+^ T Th1 and CD8^+^ T cell response than Rv1566c in mice.

Good delivery systems can facilitate the success of TB vaccine candidates, we chose the most commonly employed cationic liposome DDA in combination with immune-stimulatory factor Poly I: C, the mixture has been proved to improve immune response against the subunit vaccine in previous studies (45, 46). The strong humoral and cellular immune responses generated by Rv1566c-444, Rv1566, and Ag85B in mice indicated that the DP mixture can be used as a promising adjuvant candidate to supply the potent immunity. However, the safety of the DP mixture needs to be taken into consideration before clinical use given its cytotoxicity.

In conclusion, our study showed that the protein Rv1566c-444 enriched with T-Cell epitopes has potential as a diagnostic biomarker by evaluating T cell response to distinguish TB patients and healthy donors, and the diagnosis performance was similar to that of the whole Rv1566c protein, whilst the diagnostic sensitivity of single-antigen Rv1566c-444 was inferior to ESAT-6&CFP-10&Rv3615c used in T-SPOT.TB kit. Rv1566c-444 induced a much better cellular immune response than Rv1566c and elicited a high level of humoral response in the mouse model, providing support for further evaluation of Rv1566c-444 as vaccine antigen against MTB challenge in animal models in the form of protein mixture or fusion protein. Due to the advantage of the shorter sequence due to reducing the non-T-Cell epitope regions, the immunodominant antigen Rv1566c-444 shows great potential in developing multi-antigen diagnostic agents, multi-component subunit vaccines, and recombinant BCG vaccines for TB control.

## Data Availability Statement

The original contributions presented in the study are included in the article/supplementary material. Further inquiries can be directed to the corresponding authors.

## Ethics Statement

The studies involving human participants were reviewed and approved by the Ethics Committee of the National Institute for Communicable Disease Control and Prevention, Chinese Center for Disease Control and Prevention. Written informed consent to participate in this study was provided by the participants’ legal guardian/next of kin. The animal study was reviewed and approved by the Animal Experimental Ethical Committee of the Chinese Center for Disease Control and Prevention. Written informed consent was obtained from the owners for the participation of their animals in this study.

## Author Contributions

XiuL, XF, and KW designed the study. HL contributed to the epitope prediction. XiuL, RW, YD, ZC, NL and YY performed experiments. XiaL and GL analyzed and interpreted data. KW provided reagents. XiuL, GL, and KW wrote the manuscript. All authors contributed to the article and approved the submitted version.

## Funding

This work was financially supported by the projects of 2018ZX10731301-002 and 2018ZX10731301-002-005. The funders had no role in the study design, data collection and analysis, manuscript preparation or decision to publish

## Conflict of Interest

The authors declare that the research was conducted in the absence of any commercial or financial relationships that could be construed as a potential conflict of interest.

## Publisher’s Note

All claims expressed in this article are solely those of the authors and do not necessarily represent those of their affiliated organizations, or those of the publisher, the editors and the reviewers. Any product that may be evaluated in this article, or claim that may be made by its manufacturer, is not guaranteed or endorsed by the publisher.
